# Seminal Plasma Analysis of Oxidative Stress in Different Genitourinary Topographical Regions Involved in Reproductive Tract Disorders Associated with Genital Heat Stress

**DOI:** 10.3390/ijms21176427

**Published:** 2020-09-03

**Authors:** Monika Fraczek, Lukasz Wojnar, Marzena Kamieniczna, Malgorzata Piasecka, Kamil Gill, Michal Kups, Valentina Chopyak, Anna Havrylyuk, Jozef Nakonechnyy, Andrij Nakonechnyy, Tomasz Wozniak, Maciej Kurpisz

**Affiliations:** 1 Institute of Human Genetics, Polish Academy of Sciences, 60-479 Poznan, Poland; marzena.kamieniczna@igcz.poznan.pl (M.K.); tomasz.wozniak@igcz.poznan.pl (T.W.); 2 Clinic of Urology and Oncological Urology, Poznan University of Medical Sciences, 61-285 Poznan, Poland; lukaszwojnar@gmail.com; 3 Department of Histology and Developmental Biology, Pomeranian Medical University in Szczecin, 71-210 Szczecin, Poland; mpiasecka@ipartner.com.pl (M.P.); kamilgill@wp.pl (K.G.); michalkups1@gmail.com (M.K.); 4 Department and Clinic Urology and Oncological Urology, Regional Specialist Hospital in Szczecin, 71-455 Szczecin, Poland; 5 VitroLive Fertility Clinic in Szczecin, 70-483 Szczecin, Poland; 6 Department of Clinical Immunology and Allergology, Danylo Halytskyy Lviv National Medical University, 79008 Lviv, Ukraine; chopyakv@ukr.net (V.C.); ahavrylyuk@meta.ua (A.H.); 7 Department of Urology, Danylo Halytskyy Lviv National Medical University, 79010 Lviv, Ukraine; nyosyf@ukr.net; 8 Department of Paediatric Surgery, Danylo Halytskyy Lviv National Medical University, 79059 Lviv, Ukraine; andrurol@gmail.com

**Keywords:** genital heat stress, oxidative stress, epididymis, accessory glands

## Abstract

The pathophysiological mechanisms responsible for male subfertility/infertility caused by or complicated by genital heat stress remains unclear in many respects. Because seminal plasma creates the environment for the proper functioning of spermatozoa, in this study, we verified the associations among standard spermiograms, seminal biochemical parameters (neutral alpha-glucosidase, fructose, and citric acid) and oxidative stress markers (total antioxidant capacity, catalase activity, superoxide dismutase activity, and malondialdehyde concentration) in distinct entities associated with male infertility with and without long-time exposure to local hyperthermia. We demonstrated that men exposed to prolonged environmental or clinically recognized local heat stress in adulthood may suffer from dysregulation of seminal antioxidant components, which can be directly associated with epididymal and prostate function. The comparative analysis of the studied parameters showed numerous correlations among all biochemical parameters (particularly neutral alpha-glucosidase) with low standard semen quality in almost all the investigated infertile groups. In light of the data obtained in this originally designed study, we conclude that more attention should be paid to the epididymis and accessory gland function in subfertile and infertile men exposed to genital heat stress, especially in the context of novel treatment algorithms (targeted therapies).

## 1. Introduction

It is well known that, for effective sperm production, the male gonads require stable thermal environments at a minimum 2 °C below the temperature of the body core. Prolonged exposure of the scrotum to elevated temperature leads to disturbances in thermoregulatory mechanisms and, as a consequence, can result in the phenomenon of testicular overheating. In this context, the exposure of the testes to increased temperature is considered a risk factor for male infertility [1]. A majority of studies indicated deterioration of conventional sperm parameters in men exposed to thermogenic factors resulting in the partial or complete inhibition of spermatogenesis; however, the pathophysiology has not been fully understood. In general, two main groups of thermogenic factors can be distinguished: clinically recognized (internal) factors associated with local or systemic disease (e.g., varicocele, cryptorchidism, obesity, or febrile episodes) and environmental (external) factors related to behaviour, lifestyle and/or occupations (e.g., sitting or sleeping postures, tight clothing, hot baths, saunas, cycling, sedentary working modes, or working near high temperature sources) [2,3].

Clinical abnormalities such as varicocele and cryptorchidism are the most controversial factors; however, simultaneously, they may be the most common pathologies that may be fundamental for causing male infertility. A varicocele is an enlargement of veins of the pampiniform plexus, which under normal conditions is responsible for venous blood return from the scrotum. Patients with varicoceles often but not always demonstrate reduced fertility potential. Combinations of factors such as hyperthermia, hormonal imbalance, hypoxia, hypoperfusion, oxidative stress, increased apoptosis, and exogenous toxicants are thought to be involved in abnormal sperm spermiogram in men with this pathology [4,5]. In turn, cryptorchidism, defined as the absence of one or both testes from the scrotum, is one of the most common developmental abnormalities in boys, with a high probability of causing reproductive problems in adulthood, including infertility and testicular dysgenesis syndrome [6]. The aetiology of congenital undescended testes is mostly idiopathic and involves multiple hormonal, genetic and environmental factors [7]. Adult men with a history of cryptorchidism in childhood usually show decreased conventional sperm parameters; however, the spermiograms can be variable in both unilateral and bilateral cryptorchid individuals [8]. Despite many years of intensive research, the exact mechanisms by which varicocele and cryptorchidism impairs spermatogenesis and sperm fertilization potential remain largely unknown.

It is well known that an imbalance between pro- and antioxidant systems in semen can contribute to male infertility. The results of recent experimental prospective studies suggest that oxidative stress can also be involved in suppression of spermatogenesis caused by scrotal hyperthermia. Men exposed to transient experimental hyperthermia showed increased seminal lipid peroxidation as measured by malondialdehyde (MDA) levels [9]. There are a number of clinical reports postulating the role of oxidative stress in the pathophysiology of male infertility associated with varicocele. In patients with this pathology, an increase in levels of reactive oxygen species (ROS) [10] and/or MDA concentrations [11,12] in seminal plasma has been observed. In turn, testicular exposure to thermal shock in cryptorchidism has also been associated with concomitant oxidative stress [13]. This was confirmed by observation of elevated ROS levels in patients who had previously undergone orchidopexy [14] and in experimentally induced cryptorchidism in rodents [15,16].

The biochemical components of seminal plasma produced by the epididymis and accessory glands create an environment conducive to the proper functioning of spermatozoa. The role of seminal biochemical compounds such as neutral alpha-glucosidase (NAG), γ-glutamyltranspeptidase, fructose, zinc, citric acid and others in the pathogenesis of male subfertility/infertility has long being discussed, especially in the context of their recommendation for the use as discriminatory markers in the diagnosis of hypofunction of different topographical regions involved in reproductive tract disorders. Interestingly, previous published data have suggested that a reduction of fertilizing capacity in men with varicocele can result from damage not only testis but also epididymis demonstrating the lower levels of NAG activity concomitant with higher sperm DNA fragmentation levels and reduced sperm binding to hyaluronic acid in the patients with this pathology [17]. Nevertheless, the involvement of a thermogenic factor in modifications of the secretory function of the epididymis in varicocele has not yet been determined.

The present clinical retrospective study, for the first time, offers an integrative analysis of seminal biochemical parameters of the testis, epididymis and accessory glands as well as oxidative stress markers in distinct entities of male infertility with or without exposure to genital heat stress. Additionally, correlations between the analysed biochemical parameters and spermiogram findings were determined. Due to the multifactorial nature of varicocele and cryptorchidism, to better understand the role of thermogenic factors in these pathologies, a group of professional drivers was also included in the study. It was noted that, to the best of our knowledge, to date, there have been no clinical studies that offer similar seminal plasma quality measures in infertile adult men with history of cryptorchidism in childhood.

## 2. Results

### 2.1. Comparative Analysis of Biochemical Parameters among the Studied Groups

**Figure 1:**
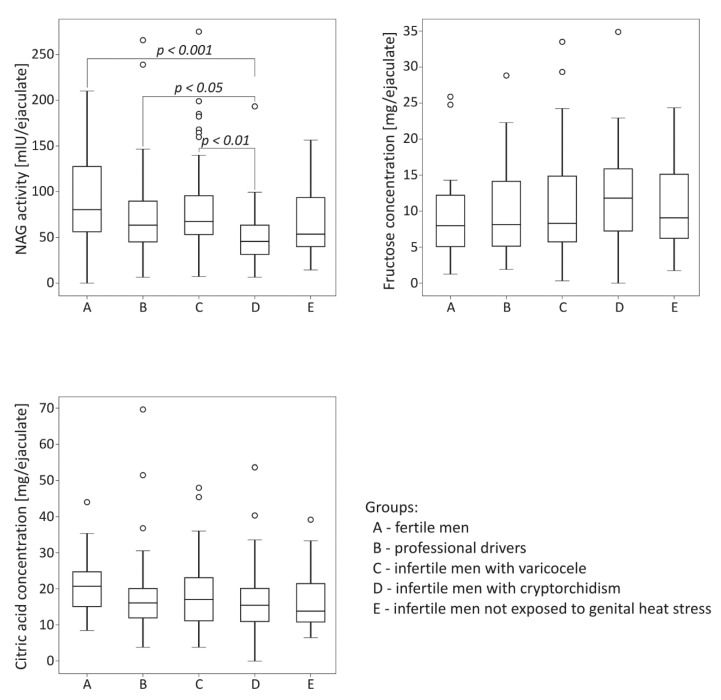
Comparison of seminal neutral alpha-glucosidase (NAG) activity, fructose concentration and citric acid concentration among the studied groups. The results are expressed as the median, Q1–Q3, and range. *p* < 0.001 calculated using the Dunn test with Holm’s correction compared to the control group; *p* < 0.01, *p* < 0.05, calculated using the Dunn test with Holm’s correction compared to the group with cryptorchidism. °—data points with values greater than Q3 + 1.5 × interquartile range.

The comparison of biochemical parameters among the studied groups is summarized in Figure 1. The activity of NAG was significantly lower in the group of infertile men with cryptorchidism than in the control fertile group (*p* < 0.001). The neutral alpha-glucosidase levels in cryptorchid men were also significantly lower than those obtained for men with varicocele and for drivers (*p* < 0.01 for the varicocele group, *p* < 0.05 for the drivers group). Regarding fructose and citric acid levels, no significant differences among the studied groups were demonstrated.

### 2.2. Comparative Analysis of Oxidative Stress Markers among the Studied Groups

**Figure 2:**
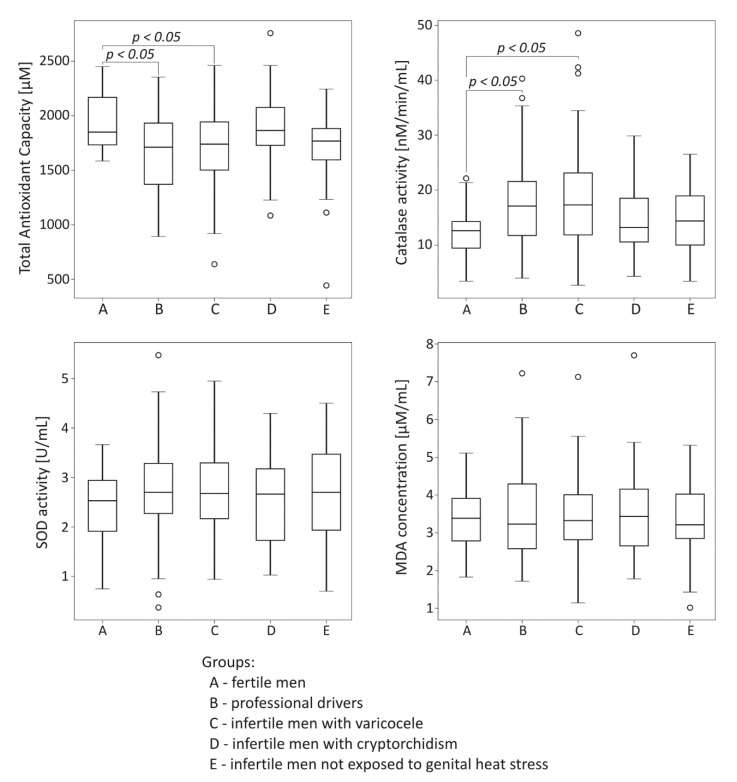
Comparison of seminal total oxidant capacity, catalase activity, superoxide dismutase (SOD) activity, and malondialdehyde (MDA) concentration among the studied groups. The results are expressed as the median, Q1–Q3, and range. *p* < 0.05 calculated using the Dunn test with Holm’s correction compared to the control group. °—data points with values smaller than Q1 − 1.5 × interquartile range or greater than Q3 + 1.5 × interquartile range.

The comparison of oxidative stress markers among the studied groups is summarized in Figure 2. Total antioxidant capacity was significantly lower both in the drivers group and the infertile men with varicocele compared to the control fertile group (*p* < 0.05). In the same groups exposed to genital heat stress, the significant reduction of TAC was accompanied by statistically significant increase in catalase activity compared to values obtained for the control fertile group (*p* < 0.05). There were no significant differences in seminal plasma SOD activity and MDA concentration among the groups under study.

### 2.3. Spearman Rank Order Correlations between Seminal Biochemical/Oxidative Stress Parameters and Standard Semen Characteristics in the Studied Groups

In Table 1, the correlation values between the biochemical parameters and the quality of the spermiogram are summarized. There were as many as seven significant correlations in the group of professional drivers. It should be noted that seminal NAG activity was positively correlated with sperm concentration, total sperm count, round cell count and semen volume. Negative correlations between NAG activity and the percentage of swollen sperm were also observed. Moreover, fructose and citric acid contents were positively correlated with semen volume. In the group of infertile men with varicocele, NAG activity showed positive correlations with both sperm concentration and total sperm count. Additionally, strong positive correlations of semen volume with fructose and citric acid levels were noted. It should be highlighted here that two similar positive correlations of fructose and citric acid with semen volume were also observed in the group of infertile men with history of cryptorchidism. These were the only correlations demonstrated in this study group. For the group of infertile men not exposed to genital heat stress, a positive relationship of NAG activity with sperm concentration and total sperm count was noted. Additionally, positive correlations of fructose or citric acid with semen volume were observed in this study group.

No associations were found between oxidative stress parameters and standard semen characteristics in the studied groups.

### 2.4. Spearman Rank Order Correlations between Oxidative Stress and Biochemical Parameters in Studied Groups

Table 2 shows the correlation values between oxidative stress and biochemical parameters in seminal plasma. In the group of drivers, a negative correlation between TAC and citric acid levels was found. A similar significant relationship was also observed in men with varicocele and in men with history of cryptorchidism in childhood. Moreover, in the group of infertile men with varicocele, the decrease in TAC was associated with the increase in NAG activity. In addition, a positive correlation between catalase activity and MDA levels was also observed. Interestingly, in the group of infertile men not exposed to genital heat stress, a strong negative correlation between SOD activity and NAG levels was demonstrated.

Additionally, in the group of professional drivers, positive correlations between all the biochemical parameters were observed. A positive correlation between fructose and citric acid was also observed in men with varicocele and cryptorchidism in childhood.

## 3. Discussion

The significance of seminal plasma biochemical quality for male fertility has not been properly recognized in many aspects. In the majority of previous published reports, biochemical components of seminal plasma between normozoospermic and pathological seminal conditions were compared, and varying results were obtained [18,19,20,21]. In the present study, we measured the principal biochemical compounds derived from the epididymis (NAG), seminal vesicles (fructose) and prostate (citric acid) in distinct entities of male infertility as well as in fertile men. What is more, the selection of the groups under study was aimed at increasing our knowledge of the pathophysiology of human infertility caused or complicated by genital heat stress.

The results obtained in the groups exposed to long-term scrotal hyperthermia, namely, professional drivers and infertile patients with varicoceles, did not show significant variations in the three biochemical markers compared to the control group as well as to the group of infertile men not exposed to prolonged thermogenic factors (Figure 1). Such findings are partially consistent with those of a previous experimental prospective study conducted on volunteers undergoing scrotal warming in a 43 °C water bath, who showed no severe changes in NAG, fructose and zinc between intermittent and consecutive transient scrotal hyperthermia [9]. On the other hand, reduction of seminal NAG activity has previously been shown in patients with varicocele, corroborating with the results of the present study [17]. Experimental models of this pathology have also revealed structural and functional changes in the epididymis of adolescent rats [22]. A lack of significant difference in seminal NAG activity between the group with varicocele and the fertile patients in the present study does not exclude the notion that varicocele-related male infertility is associated with sperm maturation disturbances (not studied here) originated during transit through the epididymal duct. Changes in patient proportions depending on the selection of men with varicocele of grades I, II and III could also influence the statistical estimates. Moreover, the clearly reduced median values observed for NAG activity in the group of drivers compared to fertile men can suggest some participation of local hyperthermia in the modification of the secretory function of the epididymis as well as in the case of varicocele.

The importance of the assessment of NAG activity in seminal plasma as a critical and non-invasive marker for evaluating the pathological changes of epididymis, especially in the context of the diagnosis of an obstruction in patients with azoospermia, has been demonstrated by a number of researchers [18,19]. In the present study, significantly lower NAG was shown in infertile men with a history of cryptorchidism in childhood than in the control group. Moreover, when compared to the group of drivers and infertile men with varicocele, NAG level in cryptorchid men was also significantly lower (Figure 1). It should be noted here that the quality of semen in adult individuals with history of cryptorchidism has been poorly addressed to date. However, few clinical studies available in the existing literature indicated the reduced sperm concentration, motility and morphology in this group of patients [8,23]. Moreover, a higher incidence of azoospermia has also been found in abnormal topographic localization of both testes and delayed surgical and/or hormonal treatment [8]. Consistent with this, in our group of infertile men with history of cryptorchidism, as many as 10 men showed no sperm in the ejaculate, while 18 revealed severe oligozoospermia (data not shown). Surprisingly, such drastically deteriorated semen quality in relation to sperm count did not correlate with the seminal NAG level in cryptorchid men, whereas such positive correlations occurred in all the remaining studied groups (Table 1). These findings may suggest that a reduction in seminal NAG levels is not always linked to a reduced sperm count and/or azoospermia caused by obstruction. This hypothesis is partly consistent with the results obtained by Lei et al. [24], who reported significantly higher NAG levels in patients with Sertoli-cell-only syndrome and severe hypospermatogenesis than in those with normal spermatogenesis or mild hypospermatogenesis. Most probably, poor semen quality of men with the history of cryptorchidism was mainly a consequence of dysregulation of homeostasis within the seminiferous epithelium that occurred at the time of testicle heating. At the present stage of research, there is no evident explanation for very low NAG levels in this group of patients, except that this could be a consequence of a direct effect of primary testis damage on the epididymis.

The relationship between the biochemical constituents of seminal plasma and standard semen characteristics is still discussed. The results of our study are partly in accordance with those of other reports demonstrating positive correlations between NAG, fructose and/or citric acid with semen volume in men with impaired fertility [25,26]. In the case of fructose and citric acid, their direct relationship was observed in all the study groups (Table 1). Such consistent results produced in this study once again confirm that the functionality of accessory glands significantly subsidizes the seminal volume. It should be emphasized that the potential effect of increased duration of ejaculatory abstinence on the studied semen parameters (especially semen volume) can be omitted here because over 92% of participants had strict 3–4-day sexual abstinence. The weak association between the function of epididymal and accessory glands and relation to sperm motility were also demonstrated by some researchers [18,20,27]. On the other hand, others did not observe such relationship [20,28,29]. We were also not able to find statistically significant correlations between sperm motility and levels of biochemical markers in the seminal fluid in any of the studied groups. The only potential relationship between the progressive movement of sperm and the fructose content was visible in the group of infertile patients with varicocele, but with borderline of statistical significance. When interpreting these findings, it should be highlighted that the design of present study could not allow us to confirm the direct regulatory effect of post-testicular glands on sperm movement. These observations once again indicate that the pathways involved in sperm motility are complex and multifactorial.

The decline in male reproductive potential that has been observed for many years was ascribed mainly to the harmful effects of the surrounding environment. One of the factors considered when explaining the pathophysiology of male infertility could be genital heat stress. Our results obtained in the group of professional drivers support the premise that local long-term exposure to thermogenic factor suggests changes in the epididymis and accessory glands with direct consequences for semen quality. This was evidenced by numerous significant moderate correlations among all the studied biochemical parameters as well as by significant correlations of the biochemical markers with sperm count (Table 1 and Table 2). Moreover, in the group of drivers, NAG activity positively correlated with the presence of round cells and negatively correlated with the percentage of the swollen spermatozoa (Table 1). These results are coincident with those of a previous experimental prospective report in which there were severe alterations in sperm membrane integrity visible in the HOS test in men exposed to transient scrotal hyperthermia [9]. Considering the fact that immature germ cells in semen are generally an indication of the disorder of spermatogenesis, the comparative analysis in the present study suggests that scrotal hyperthermia can be associated with sperm maturation disturbances occurring in both the testis and the epididymis.

Since spermatozoa are characterized by high susceptibility to peroxidative damage, semen contains abundant antioxidant system which include enzymatic and nonenzymatic factors for effective protection of spermatozoa against the ROS attack. Some authors consider the total antioxidant capacity (TAC) in seminal plasma to be a principal measurement of redox status that may disturb fertility [30]. In the present study, median TAC was found to be significantly lower in drivers as well as infertile men with varicocele than in the control group (Figure 2). These data suggest that men exposed to prolonged environmental or clinically recognized local active heat stress can be threatened by the harmful effects of oxidative stress. The results obtained in our study also indicated a deregulation of enzymatic antioxidant defence system in seminal plasma of men exposed to the active thermogenic factor. In fact, the activity of catalase was significantly greater in the group of drivers and the group of infertile patients with varicocele than in the control group (Figure 2). Such results are consistent with those of reports in which authors demonstrated increased catalase activity in infertile men with varicocele [31]. However, in contrast to these reports, Abd-Elmoaty et al. [32] and Mostafa et al. [12] observed decreased activity of seminal enzymes of antioxidant defence system in this pathology. Moreover, the reduction of enzymatic activity intensified with increasing varicocele grade. Studies carried out on experimental models of cryptorchidism [33,34] also revealed decreased activity of antioxidant enzymes in gonads, although to date, it has not been confirmed in ejaculates of adult men with histories of cryptorchidism. The results obtained in this study regarding parameters of seminal antioxidative defence system clearly suggest that redox imbalance in semen occurs during active scrotal overheating. We can speculate that the activation of catalase in these cases could be a natural response to oxidative stress, probably aimed to protect sperm cells from peroxidative damage; however, due to the overall reduced TAC, this cannot be an effective line of defence. This hypothesis is supported by the positive correlation observed between catalase activity and MDA concentrations in men with varicoceles (Table 2).

Oxidative stress is known to be implicated in a variety of pathophysiological events that cause male infertility. Although majority of research (including present study) on heat-induced oxidative stress and ROS-scavenging mechanisms involving antioxidants are conducted in semen, it should be mentioned that biological effects of heat-induced oxidative stress in reproductive organs are quite complex and they can differ from those observed in the ejaculates [35]. There are some premises that heat stress response of male gonad, which is characterized by high expression of the testicular antioxidant system and by the induction of a set of heat shock proteins (HSPs) can also be regulated by hormonal stimulation. Early in vitro experimental studies and animal models of cryptorchidism designed to study the effect of heat stress on the testis have demonstrated that oxidative stress is a major cause of heat-induced germ cell death, which can be accompanied by alterations in Sertoli and Leydig cells function followed by changes in testicular endocrine system [36,37,38]. Interestingly, recent experimental data have also suggested that the possible mechanism of heat stress damaged spermatogenesis with affected semen quality can be associated with a reduction of the sensitivity of receptor androgen to testosterone caused by the high expression of HSP70 [39]. Contrary to the experimental data, the hypothesis of varicocele impairment in Leydig cell function and decrease in androgen levels has not been firmly confirmed [40,41]. Moreover, recent clinical data regarding benefits of microsurgical varicocelectomy have showed improvement in sperm parameters without changes in serum as well as intratesticular testosterone [42]. It is possible that under chronic heat stress conditions human Leydig cells adapt to heat stress and maintain steroid production, which was suggested in some experimental studies [37]. Furthermore, it cannot be excluded that oxidative stress, especially antioxidant profile within the testicular microenvironment, can be involved in heat-induced interstitial damage in male gonad and its steroid metabolic pathway [35].

The level of lipid peroxidation in semen based on the MDA concentration in seminal plasma was also evaluated in this study. Surprisingly, regarding this parameter, there were no significant differences among the studied groups (Figure 2). These findings contradict those of other clinical reports, in which statistically higher levels of MDA in men with varicocele were observed in both seminal plasma [43,44] and testes [45]. To date, no similar results have been found in groups of infertile men with a history of cryptorchidism. However, Imamoğlu et al. [46] demonstrated an increase in MDA concentration in the blood of cryptorchid boys. When interpreting the results obtained in this study, it is necessary to explain that only a small number of products of lipid sperm membrane peroxidation is shed to seminal plasma, while the remainder is bound to sperm membranes [47]. Therefore, it cannot be excluded that intracellular MDA levels, which were not determined in this or in other studies, were higher in the examined samples of men exposed to local heat stress. In the light of these data, MDA concentration in seminal plasma appears as rather poor diagnostic marker of male infertility, in contrast to the opinion of some authors [43].

One of the specific objectives of the research aims undertaken in this study was an attempt to determine the principal correlations between level of seminal oxidative stress intensity and level of biochemical seminal compounds in the studied groups. Our data revealed such a direct relationship and showed specific correlations in males exposed to genital heat stress both in childhood and adulthood. In the groups of drivers, infertile men with varicocele and those with history of cryptorchidism, similar negative correlations of TAC with citric acid content were observed. Additionally, in the varicocele group, seminal TAC correlated with NAG (Table 2). These results suggest direct impact of epididymal and prostate function on seminal quality in men exposed to long-term scrotal hyperthermia, especially in the context of non-enzymatic antioxidant agents of seminal plasma. This hypothesis is clearly in agreement with the experimental data obtained by Ahmad et al. [48] who observed decreases in oxidative stress induced by temperature in the presence of ascorbic acid. In this context, the correlations with citric acid levels observed in this study may shed light on the role of zinc (another typically representative agent of prostatic secretory function) in maintaining good sperm quality in men exposed to local heat stress, especially with regard to its beneficial role on the sperm membrane fluidity and stability as well as chromatin maturity [49]. A number of animal model studies proved that dietary zinc supplementation reduced oxidative stress in the testis [50] and epididymis [51] induced by local heat exposure. It was further reported that an adverse effect of scrotal hyperthermia on sperm parameters in mice could be avoided by long-term zinc administration [52]. Regardless of these promising results, further studies are needed to elucidate the associations between seminal non-enzymatic reducing agent concentrations and sperm fertilizing potential in men exposed to both external or internal thermogenic factors.

Superoxide dismutase is a principal component of the antioxidative defense system in the male reproductive tract. Some studies indicated the role of seminal SOD as a predictive factor for male infertility and detected a positive correlation of seminal SOD activity with sperm concentration, motility, viability and/or morphology [53,54]. In contrast to these studies, others have suggested that seminal SOD activity was not associated with reduced semen quality [55]. In the present study, there were no differences found in the activity levels of SOD in semen between fertile and infertile men (Figure 2). It should be emphasized, however, that in our previously published reports, diminished SOD activity in seminal plasma appeared to be more likely associated with the particular type of spermiogram [56]. We cannot exclude the possibility that SOD is acting in microenvironment of the other antioxidants of which capacity creates feedback with SOD activity; therefore, pathological spermiogram may reflect current redox status as a result of such interplay. Thus, the situation with regard to this enzyme measured in seminal plasma is largely unexplained in specific entities of male infertility, also in the context of the origin of SOD in semen. In our study, SOD negatively correlated with NAG levels in the group of infertile men not exposed to genital heat stress (Table 2). Such data are consistent with previous reports, in which the authors stated that SOD is largely secreted from the other accessory glands with no substantial testicular or epididymal contribution [57,58].

Some limitations of our study should be briefly mentioned. First, the study was conducted on 226 participants, which is a relatively small study group. It is possible that if we had increased the number of samples, the obtained results would have achieved higher statistical significance. Second, the study participants did not have scrotal temperature measurement because this is not a common andrological practice and the precise devices for taking scrotal temperature are not widely available. Nevertheless, the design of this study, the inclusion of distinct groups of men exposed to both external or internal thermogenic factors and the strict exclusion criteria of the study allowed us to reduce selection bias. Third, the study was a part of a multi-centre research project and variations in laboratory measurements of standard semen parameters, especially sperm motility, could limit the validity of some data. Nevertheless, all researchers responsible for standard semen analysis were experts in sperm biology and male infertility. Fourth, the study did not provide the full picture of the impact of the post-testicular glands on antioxidant activity of seminal plasma, limiting it only to the enzymatic component and TAC.

In conclusion, in this originally designed study, we demonstrated that the biochemical status of seminal plasma may be responsible for low sperm quality in men exposed to both environmental and internal genital heat stress. Dysregulation of seminal antioxidant components can be principally associated with the epididymal and prostate functions. Moreover, each group of these parameters forms a unique micro-pattern in semen characteristics for the particular entities of male infertility. The obtained data may hopefully provide valuable insight into the molecular mechanisms that help us understand the pathophysiology of male infertility caused or complicated by genital heat stress, which would establish new diagnostic biomarker(s) and treatment (targeted therapy) algorithms for male subfertility/infertility associated with scrotal hyperthermia.

## 4. Materials and Methods

The study was a part of a multi-center research project which was approved by the Local Bioethical Committee at the Poznan University of Medical Sciences, Poland (ethical authorization number: 730/16, 16 June 2016). Written informed consent was obtained from all subjects. The information about the participants was anonymously coded to protect personal data. All experiments were performed in accordance with relevant guidelines and regulations.

### 4.1. Male Participants

The male participants included 226 patients and volunteers of reproductive age (22–40 years) who were recruited from the Andrology Outpatient Clinics in Poznan, Szczecin and Lviv as well as via traditional and social media advertising. All participants were subjected to a routine infertility work-up that included their medical history as well as andrological and ultrasound examinations. Men who had failed to achieve conception for at least 12 months without any apparent reason on the partner’s side were classified as infertile. Men who had at least one offspring at two years maximum were classified as fertile. The varicoceles were diagnosed when dilation of the vessels of the pampiniform plexus were ≥3 mm in diameter. Additionally, the men were asked to complete a questionnaire containing a series of questions on general health, genitourinary diseases, working conditions and lifestyle. On the basis of the collected clinical and survey data, the men were qualified to one of the following five research subgroups: (A) group of fertile men not exposed to prolonged thermogenic factor; serving as the control group (n = 21); (B) group of professional drivers; minimum 2 years in profession (n = 52); (C) group of infertile men treated for cryptorchidism in childhood (n = 50); (D) group of infertile men with varicocele (n = 71); (E) group of infertile men not exposed to prolonged thermogenic factor (n = 32). The main exclusion criteria included the factors that may additionally affect redox status, i.e., age over 40 years, body mass index ≥ 30, smoking, drug usage, co-existing systemic or local active inflammatory process, and double thermogenic factors. All semen samples were collected for the study before any medical treatment (surgical interventions, medicines, supplements).

### 4.2. Semen Collection and Processing

All semen samples were obtained by masturbation after 3–5 days of sexual abstinence. Within 60 min of ejaculation and liquefaction, standard semen analysis was performed manually according to the World Health Organization 2010 criteria [59]. In brief, sperm concentration was assessed with the improved Neubauer haemocytometer (Paul Marienfeld, Lauda-Königshofen, Germany). Sperm motility was evaluated using the standard grading system: progressive motility, non-progressive motility and immotility. To assess sperm viability both eosine staining and hypo-osmotic swelling (HOS) test were performed. Sperm morphology (including teratozoospermia index–TZI) was evaluated using the Papanicolaou staining method. The Endtz test was used to count and differentiate peroxidase-positive leukocytes and peroxidase-negative round cells (other round cells) in the semen samples (LeucoScreen kit, FertiPro N.V., Beernem, Belgium). The conventional semen parameters for all the studied groups have been summarized in Table 3. Spermatozoa from collected samples were separated from seminal plasma by centrifugation at 1800 rpm for 7 min. Seminal plasma was centrifuged again at 3500 rpm for 5 min; then divided into aliquots and stored at −75 °C for the determination of oxidative stress and biochemical parameters. Seminal plasma collected in Szczecin and Lviv were transported on dry ice to the Andrology Laboratory in Poznan. All the samples were stored for a maximum of 3 months before being analysed.

### 4.3. Determination of Biochemical Parameters in Semen Samples

Neutral α-glucosidase, fructose and citric acid were determined using commercially available test kits from FertiPro N.V. (FertiPro N.V., Beernem, Belgium). For all parameters, duplicates of each standard and unknown sample were read on 96-well microplates compatible with a spectrophotometric plate reader (ELx808, Bio Tek Instruments, Inc. Winooski, VT, USA), and the mean of the two optical density (OD) values was taken.

#### 4.3.1. Neutral Alpha-Glucosidase Activity Measurement in Seminal Plasma

An improved NAG assay with the usage of glucose as an enzyme inhibitor for background correction was performed [60]. The principle of the test was based on the conversion of 4-nitrophenyl-α-D-glucopyranoside into 4-nitrophenol and α-D-glucopyranoside. The yellow colour of 4-nitrophenol in sample reaction and sample inhibitor was measured spectrophotometrically at a wavelength of 405 nm for each semen sample. Enzyme activity was calculated on the basis of corrected OD values for both reaction and inhibitor as well as on linear regression curve for standard samples. The results were expressed as mIU/ejaculate.

#### 4.3.2. Fructose Concentration Measurement in Seminal Plasma

The principle of the test was based on the reaction of fructose with indole in the presence of HCl at 37 °C. The absorbance of a coloured complex was measured spectrophotometrically at a wavelength of 450 nm. The fructose content in seminal plasma was calculated by comparing the absorbance against a standard curve. The results were expressed as mg/ejaculate [59].

#### 4.3.3. Citric Acid Concentration Measurement in Seminal Plasma

The principle of the assay was based on the formation of complex of citrate and Fe^3+^ ions. The intensity of a yellow colour was measured spectrophotometrically at a wavelength of 405 nm. The results were calculated based on the absorbance of standard and were expressed as mg/ejaculate [61].

### 4.4. Determination of Oxidative Stress Parameters in Semen Samples

Total antioxidant capacity (TAC), superoxide dismutase (SOD) activity and catalase activity were determined with commercially available test kits from Cayman Chemical (Cayman Chemical, Ann Arbor, MI, USA). The OXISelect TBARS Assay Kit (Cell Biolabs Inc., San Diego, CA, USA) was used for malondialdehyde (MDA) quantification. Each standard and unknown sample were determined in two repetitions. The absorbance was measured in a spectrophotometric plate reader (ELx808, Bio Tek Instruments, Inc. Winooski, VT, USA).

#### 4.4.1. Total Antioxidant Capacity (TAC) Measurement in Seminal Plasma

Before determination of TAC, seminal plasma was diluted (1:9) by with TAC Assay kit assay buffer. The principle of the assay was based on ability of antioxidants to inhibit oxidation of the 2,2′-azinodisulfinate 3-ethylbenztiazoline (ABTS^®^) to ABTS^®+^ radical cation, which was compared with that of Trolox, a water-soluble tocopherol analogue. Suppression of the absorbance of blue-green ABTS^®+^ was measured spectrophotometrically at a wavelength of 405 nm. The results were reported as µM of Trolox equivalent [30].

#### 4.4.2. Superoxide Dismutase (SOD) Activity Measurement in Seminal Plasma

Before determination of SOD activity, seminal plasma was diluted (1:2) with SOD Assay kit sample buffer. The principle of the assay was based on the reaction of xanthine and xanthine oxidase to generate superoxide anion (O_2_^−^) which then reacted with tetrazolium salts to form red formazan dye. The degree of inhibition of this reaction was measured spectrophotometrically at a wavelength of 450 nm. The SOD activity in seminal plasma was calculated on the basis of the linear regression curve for standard samples. The results were finally expressed as U/mL [62].

#### 4.4.3. Catalase Activity Measurement in Seminal Plasma

The principle of the assay was based on the reaction of catalase with H_2_O_2_ and methanol to generate formaldehyde which then reacted with chromogen (Purpald; 4-amino-3-hydrazino-5-mercapto-1,2,4-triazole). The intensity of purple colour was measured spectrophotometrically at a wavelength of 540 nm. Formaldehyde content was calculated on the basis of the linear regression curve for standard samples. The results were finally expressed as nM/min/mL [63].

#### 4.4.4. Malondialdehyde (MDA) Concentration Measurement in Seminal Plasma

The principle of the assay was based on the formation of a colour complex of lipid peroxidation products with thiobarbituric acid (TBA) at high temperature and acidic environment. The chromogen was then extracted using n-butanol (1:1, *v*/*v*). The absorbance of the butanol fraction was measured spectrophotometrically at a wavelength of 532 nm. The MDA content in seminal plasma was read directly from the MDA standard curve. The results were expressed as µM/mL. Water served as the blank control sample [64].

### 4.5. Statistical Analysis

The data were analysed using the Python 3 with Pandas (https://pandas.pydata.org/), Matplotlib (https://matplotlib.org/), SciPy (https://www.scipy.org/) and scikit-posthoc (https://pypi.org/project/scikit-posthocs/) libraries. The distribution of the results was evaluated using the Shapiro-Wilk test. Statistical differences among the studied subgroups were determined by non-parametric Kruskal–Wallis test. In the post-hoc analysis, the Dunn test with Holm’s correction was applied. Correlations were assessed using the Spearman rank test. *p*-values < 0.05 were considered to be significant.

## Figures and Tables

**Table 1:** Spearman rank order correlations between biochemical parameters and standard semen characteristics in the studied groups.

Variables	R (Spearman)	*p* Level
Group of professional drivers		
NAG vs. sperm count/mL	0.322	<0.05
NAG vs. sperm count/ejaculate	0.440	<0.01
NAG vs. HOS test	−0.344	<0.05
NAG vs. round cells	0.330	<0.05
NAG vs. semen volume	0.420	<0.01
Fructose vs. semen volume	0.767	<0.001
Citric acid vs. semen volume	0.596	<0.001
Group of infertile men with varicocele		
NAG vs. sperm count/mL	0.401	<0.001
NAG vs. sperm count/ejaculate	0.419	<0.001
Fructose vs. semen volume	0.686	<0.001
Citric acid vs. semen volume	0.689	<0.001
Group of infertile men with cryptorchidism		
Fructose vs. semen volume	0.491	<0.001
Citric acid vs. semen volume	0.415	<0.01
Group of infertile men not exposed to genital heat stress		
NAG vs. sperm count/mL	0.565	<0.01
NAG vs. sperm count/ejaculate	0.580	<0.001
Fructose vs. semen volume	0.836	<0.001
Citric acid vs. semen volume	0.524	<0.01

Abbreviations: HOS test—hypo-osmotic swelling test; NAG—neutral alpha-glucosidase

**Table 2:** Spearman rank order correlations between biochemical and oxidative stress parameters in the studied groups.

Variables	R (Spearman)	*p* Level
Group of professional drivers		
TAC vs. citric acid	−0.309	<0.05
NAG vs. fructose	0.500	<0.001
NAG vs. citric acid	0.379	<0.01
Fructose vs. citric acid	0.342	<0.05
Group of infertile men with varicocele		
TAC vs. NAG	−0.371	<0.01
TAC vs. citric acid	−0.307	<0.01
Catalase vs. MDA	0.353	<0.01
Fructose vs. citric acid	0.364	<0.01
Group of infertile men with cryptorchidism		
TAC vs. citric acid	−0.383	<0.01
Fructose vs. citric acid	0.366	<0.05
Group of infertile men not exposed to genital heat stress		
SOD vs. NAG	−0.582	<0.001

Abbreviations: MDA—malondialdehyde; NAG—neutral alpha-glucosidase; SOD—superoxide dismutase; TAC—total antioxidant capacity

**Table 3:** Spermiogram parameters in the studied groups.

Standard Semen Parameter	Control Group (Fertile Men) (n = 21)	Group of Professional Drivers (n = 52)	Group of Infertile Men with Varicocele (n = 50)	Group of Infertile Men with Cryptorchidism (n = 71)	Group of Infertile Men not Exposed to Genital Heat Stress (n = 32)
Volume [mL]	2.95 ± 1.09	3.00 ± 1.05	3.40 ± 1.38	3.50 ± 1.40	3.30 ± 0.99
pH	8.00 ± 0.32	8.00 ± 0.31	8.00 ± 0.27	8.00 ± 0.29	8.00 ± 0.25
Sperm concentration [×10^6^/mL]	90.98 ± 52.30	20.90 ± 21.12	18.00 ± 19.82	3.25 ± 15.42	18.15 ± 30.66
Total number of spermatozoa [×10^6^/ejaculate]	272.10 ± 137.29	51.65 ± 78.83	52.50 ± 65.66	15.00 ± 49.75	57.20 ± 87.35
Progressive motility of spermatozoa [%]	52.50 ± 9.15	39.00 ± 14.54	32.00 ± 14.22	37.00 ± 13.98	33.00 ± 12.32
Total sperm motility [%]	63.00 ± 8.75	48.50 ± 13.95	46.00 ± 14.54	44.00 ± 13.71	44.00 ± 13.65
Sperm viability [%]	73.50 ± 8.50	68.50 ± 9.75	70.00 ± 12.76	65.00 ± 10.61	65.00 ± 15.58
Spermatozoa with normal morphology [%]	5.00 ± 2.59	1.50 ± 1.45	2.00 ± 1.44	1.00 ± 1.19	2.00 ± 1.17
Peroxidase-positive leukocytes [×10^6^/mL]	0.14 ± 0.40	0.17 ± 0.32	0.14 ± 0.73	0.10 ± 0.31	0.08 ± 0.59
Other round cells [×10^6^/mL]	0.80 ± 1.23	0.75 ± 1.43	0.74 ± 1.00	0.41 ± 0.49	0.63 ± 0.87
HOS test [%]	74.00 ± 5.00	68.00 ± 12.60	65.00 ± 12.69	64.00 ± 12.31	62.50 ± 14.00

Note: The results are expressed as median ± average deviation.
